# Exploring the impact of tonal inventory on speech perception across languages: a study of MMN responses in tonal language speakers

**DOI:** 10.3389/fpsyg.2024.1394309

**Published:** 2024-09-11

**Authors:** Chun-Hsien Hsu, Tong-Hou Cheong, Wen-Jun Huang

**Affiliations:** ^1^Institute of Cognitive Neuroscience, National Central University, Taoyuan, Taiwan; ^2^Department of Hakka Language and Social Sciences, National Central University, Taoyuan, Taiwan

**Keywords:** MMN, lexical tones, tonal language, phonological inventory, speech perception

## Abstract

Previous research on the perception of segmental features of languages has established a correlation between the phoneme inventory of a language and its speakers’ perceptual abilities, as indexed by discrimination tasks and Mismatch Negativity (MMN). Building on this background, the current study elucidated the relationship between perceptual ability and tonal inventory by utilizing two tonal languages. Two groups of participants were included in the present experiment: Mandarin speakers and Hakka-Mandarin speakers. Onset latency analysis revealed a significant difference in the Mandarin syllable condition, with Hakka-Mandarin speakers demonstrating earlier MMN latency than Mandarin speakers. This suggests a more efficient auditory processing mechanism in Hakka-Mandarin speakers. Both groups, however, showed similar MMN latency in the Hakka syllable condition. The interaction between language background and syllable type indicates that other factors, such as syllable sonority, also influence MMN responses. These findings highlight the importance of considering multiple phonemic inventories and syllable characteristics in studies of tonal perception.

## 1 Introduction

In language comprehension, speech perception is a critical skill involving the discrimination of both segmental and suprasegmental features. Research utilizing electroencephalography (EEG) has demonstrated that the size of a phoneme inventory can significantly influence the amplitude of the mismatch negativity response (MMN; Brunellière et al., 2011; Hacquard et al., 2007; Zhang et al., 2005). MMN is a unique ERP used to detect distinguishable changes in acoustic features within a stream of sound and is not influenced by attention (Näätänen et al., 1993, 1997). It is assumed that MMN activity indexes an automatic, pre-attentive auditory processing mechanism, and it varies depending on several factors, including the participants’ language experiences and acoustic features of the stimuli. Hacquard et al. (2007)’s study demonstrated that speakers of languages with larger vowel inventories (like French) showed larger MMN amplitudes compared to speakers of languages with smaller vowel inventories (like Spanish). This indicates that participants’ language backgrounds, and particularly the size of their vowel inventories, influence how they perceive vowel changes. Their finding was consistent with Teles and Huey (2020)’ demonstration that speakers of languages with larger vowel inventories (e.g., English or French) would expand the space of vowel dispersion relative to speakers with smaller vowel inventories (e.g., Spanish), such that they would produce the target phonemes acoustically far away from one another. Taken together, the effects of phoneme inventory size on speech production and perception imply speakers of languages with complex vowel systems would exhibit enhanced perceptual sensitivity to vowels compared to those with simpler vowel systems.

While existing studies have explored the role of segmental features in both tonal and non-tonal languages, there is the question of whether the organization of tonal representation can have an effect on speech perception. The accurate perception of suprasegmental information is also crucial in language comprehension, as it can provide listeners with important linguistic cues, such as affective-prosodic cues and prosodic phrasing. This is particularly relevant in tonal languages, such as Chinese and Thai, where pitch differences are used to differentiate words (Chandrasekaran et al., 2007; Huang and Johnson, 2011). Therefore, this study aimed to explore whether the perception of lexical tones is processed in the same way regardless of the size of the tonal inventory of participants’ language. This prediction was based on the finding that the size of the phonological inventory correlates with the ability to produce and perceive spoken sounds in monolinguals and bilinguals.

Instead of using behavioral measurements, the preset study applied the event-related potentials (ERP) method and measured the MMN responses to syllables. Numerous studies of speech perception have focused on MMN. The MMN paradigm typically involves a rapidly presented stream of repeated standard sounds occasionally interrupted by rare deviant sounds. MMN activity can be measured by comparing ERP responses to the deviant sound with those to the standard sound or by comparing ERP responses to the deviant sound in an MMN experiment with those to the same sound in an equal-probability control block (Jacobsen and Schröger, 2001). In addition to the amplitude of ERP activity, ERP studies also have demonstrated that the delay in the latency of MMN activity was associated with the insufficiency of phonological perception during the processing of linguistic stimuli (Alonso-Búa et al., 2006; Cheng et al., 2021; Zhang et al., 2005). For example, native Japanese speakers often struggle to differentiate between the English phonemes /r/ and /l/, both of which are mapped to Japanese /l/. Zhang et al. (2005) used magnetoencephalography (MEG) to record MMN in response to /r/ and /l/ sounds in native Japanese and native American English listeners. The study found that native Japanese listeners were less sensitive to the phonemic /r-l/ difference than native American English listeners, and their MMN amplitudes and latency were significantly smaller and longer, respectively.

**Figure 1:**
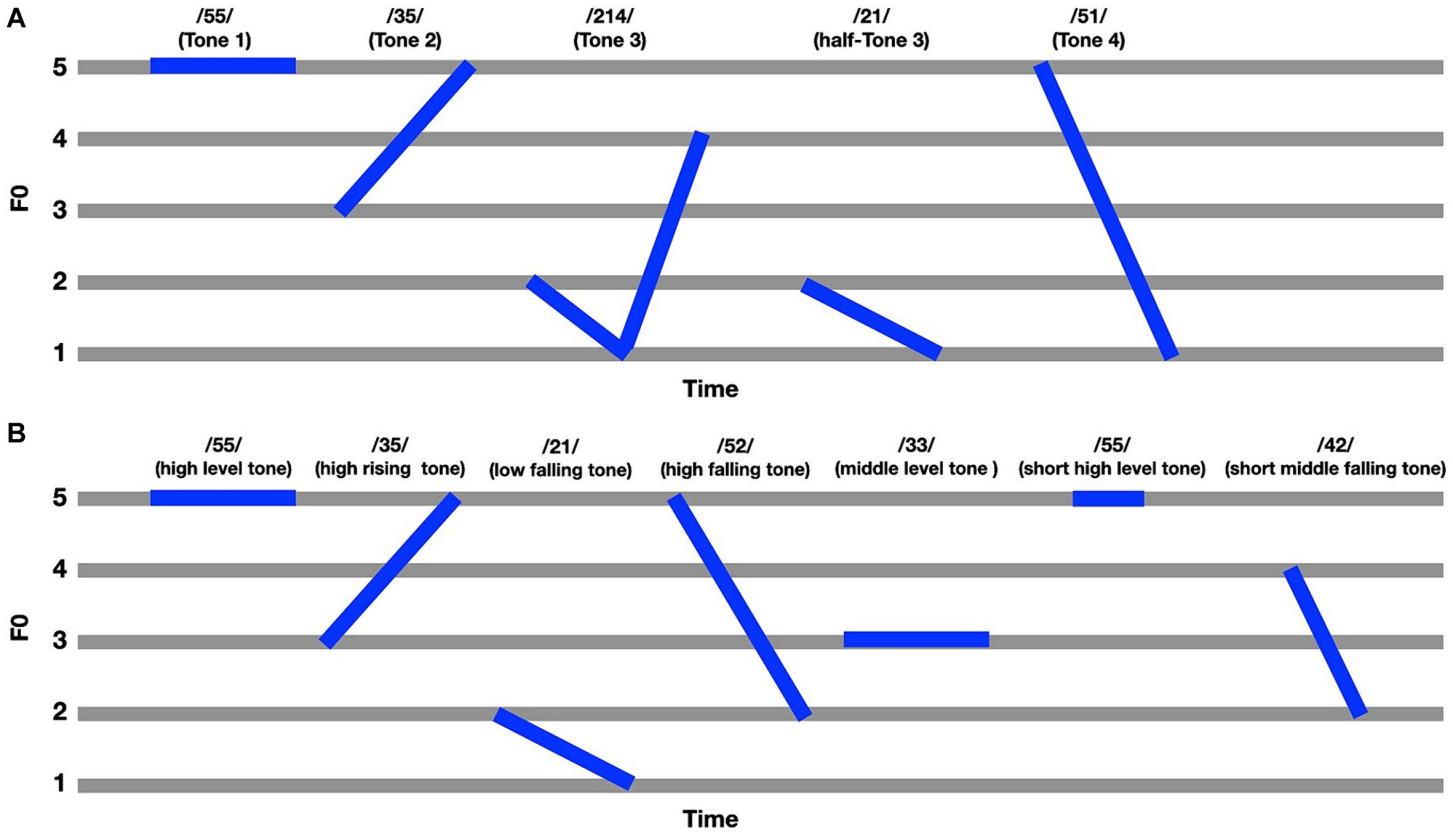
Representation of lexical tones in Mandarin Chinese **(A)** and Hailu Hakka **(B)** utilizing a five-level scale for tone marks (Chao, 1968). The digits on the left indicate the pitch level, where 1 corresponds to the lowest and 5 to the highest pitch. **(A)** The lexical tones of Mandarin using the five-level scale as described by Duanmu (2007); **(B)** The lexical tones of Hailu Hakka according to the study by Huang and Yu (2022).

In the case of tonal inventory, the five-scale tone representation scheme is a method used to describe the pitch contour of lexical tones in tonal languages (Chao, 1968). This scheme employs a numerical scale from 1 to 5, where each number represents a specific pitch level, with 1 being the lowest and 5 the highest. Using this scheme, the tonal contour of a syllable can be described by a sequence of these numbers (Figure 1A). For example, Mandarin Chinese syllables have four lexical tones (Duanmu, 2007; Li et al., 2023): the high level tone (Tone 1, or 55-tone, according to the five-scale tone representation scheme), high rising contour tone (Tone 2; 35-tone), low falling-raising contour tone (Tone 3; 214-tone), and high falling contour tone (Tone 4/ 51-tone). In addition to the full Tone 3 (214-tone), the half-Tone 3 (21-tone), a reduced form of the 214-tone frequently used in natural speech, is an allophonic variant of the traditional 214-tone (Fu and Lee, 2022; Lu and Lee-Kim, 2021; Zhang and Lai, 2010). Chandrasekaran et al. (2007) used two experimental blocks with different tonal contrasts. In one block, the participants frequently heard the syllable /yi/ with 55-tone and occasionally heard /yi/ with 214-tone. In the other block, the standard stimulus was /yi/ with 35-tone. The results showed that native Mandarin speakers’ MMN responses to the 55/214 contrast were larger than their MMN responses to the 55/35 contrast, indicating that MMN amplitudes are correlated with the acoustic similarity between pairs of standard and deviant sounds. Native English speakers’ MMN did not demonstrate the effect of tonal contrast on MMN. Furthermore, while native Mandarin speakers’ MMN to the 55/214 contrast was larger than native English speakers’ MMN to the 55/214 contrast, there was no significant group difference in the 55/35 contrast. These findings suggest that native tonal speakers are more sensitive to the height dimension than to the contour dimension, and that nontonal speakers’ pitch perception does not appear to be significantly dependent on the height versus contour distinction.

As previously mentioned, it is not clear whether the size of a tonal language’s inventory would affect the ability of speakers to perceive and differentiate pitch. To address this gap, the current study aimed to compare MMN responses to tonal contrasts in two groups of speakers: Mandarin Chinese and Hakka(Hailu)-Mandarin Chinese bilingual speakers to investigate whether there is a correlation between the complexity of the tonal system and the acuity of pitch perception. Hailu Hakka and Mandarin Chinese have similar basic vowels and word-initial consonants, and Hailu Hakka has more liaison consonants. They both have three syllable structures, including CV (Consonant-Vowel), CVV (Consonant-Vowel-Vowel) and CVC (Consonant-Vowel-Consonant) forms. Regarding the lexical tones of Hailu Hakka, there are seven distinct tones (Huang and Yu, 2022). Among these, four tones are similar to the lexical tones of Mandarin (Figure 1B): the high-level tone (55-tone), which corresponds to Mandarin’s Tone 1; the high rising contour tone (35-tone), which resembles Mandarin’s Tone 2; the low falling tone (21-tone), akin to the half-Tone 3 in Mandarin; and the high falling tone (52-tone), which parallels Mandarin’s Tone 4. According to the review by Huang and Yu (2022), the low falling tone in Hakka has been coded as both a 31-tone and a 21-tone in different studies. This variation arises due to the different methods of normalization and analysis employed across these studies. To ensure consistency and avoid confusion, the low falling tone in Hakka was referred to as the 21-tone in the present study. Additionally, Hakka has unique tones that differentiate it from Mandarin: the middle-level tone (33-tone); the short high-level tone (55-tone), which is similar to Mandarin’s Tone 1 but shorter in duration; and the short middle falling tone (42-tone), characterized by a mid-level pitch that falls to a lower pitch with a shorter duration. Building on the findings of Hacquard et al. (2007) regarding the impact of vowel inventory size on perceptual ability, one could posit that a similar correlation exists between the complexity of a tone system in tonal languages and the perceptual abilities of its speakers. That is, the additional tones in Hailu Hakka suggests a more complex tonal system, potentially indicating that speakers of Hailu Hakka have a more nuanced perceptual process sensitive to dynamic changes in tone. Therefore, one might expect that Mandarin participants may be less sensitive to the tonal changes as compared with Hakka-Mandarin speakers and exhibit reduced or delayed MMN response.

## 2 Materials and methods

### 2.1 Participants

A total of 17 native Mandarin speakers and 16 Hakka-Mandarin bilinguals (aged 18–30 years) were recruited to participate in the MMN experiment. All participants spoke Taiwanese Mandarin. Mandarin speakers were undergraduate and graduate students at National Central University. They majored in engineering, earth science, life science, and English or French literature. All Hakka-Mandarin bilinguals were raised in communities where Hailu Hakka and Mandarin Chinese were the primary languages of daily conversation and were pursuing undergraduate or graduate studies at the College of Hakka Studies at National Central University, where Hailu Hakka served as the main language of instruction and classroom discourse. All of the participants had normal hearing and normal or corrected-to-normal vision. The current study was approved by the Human Subject Research Ethics Committee of National Taiwan University.

### 2.2 Stimuli

**Figure 2:**
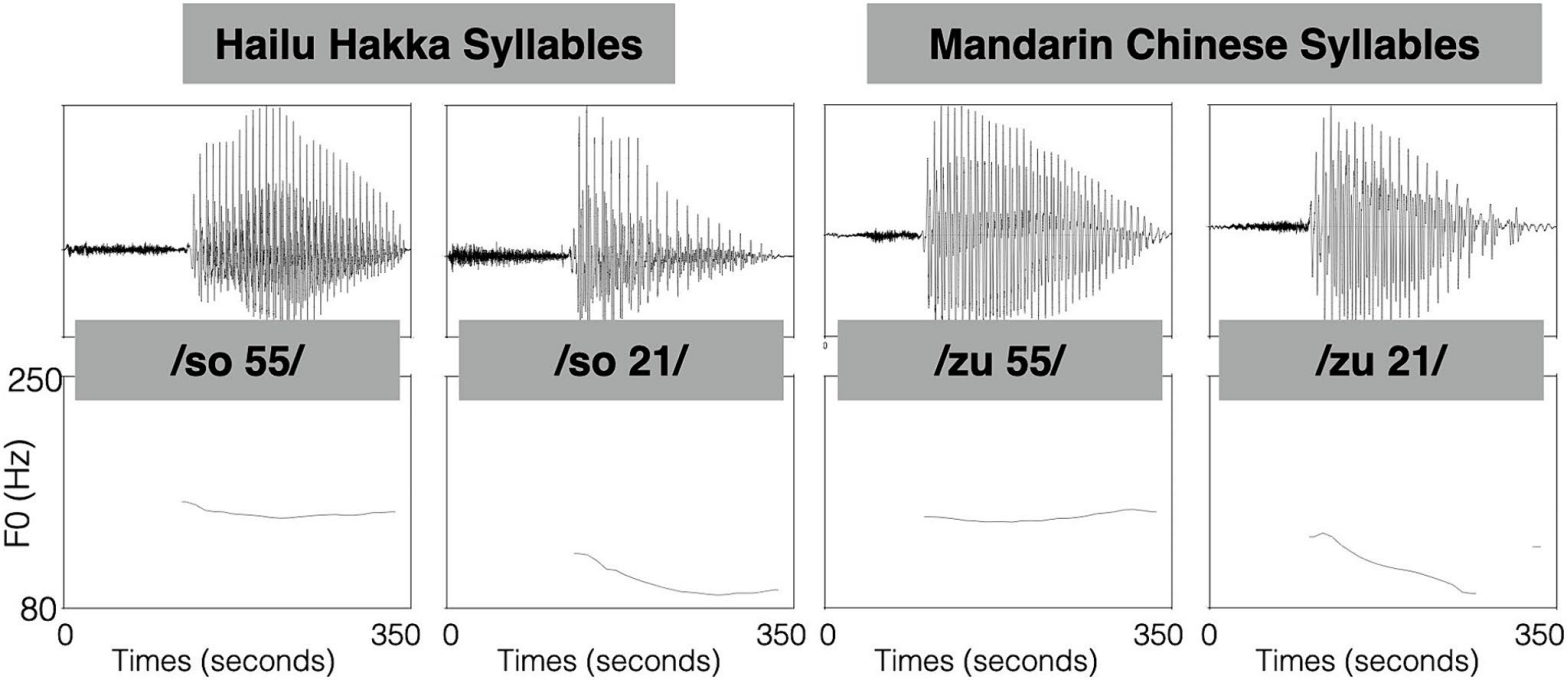
Speech waveforms and F0 contours of stimuli.

The experiment was conducted using two distinct sets of speech stimuli. The first set consisted of two Mandarin syllables, specifically /zu/ with 55-tone and 21-tone. The second set of stimuli consisted of the Hakka syllable /so/ with 55-tone and 21-tone. The selected Mandarin syllables are real words in Mandarin and are not words or morphemes in Hailu Hakka. The selected Hakka syllables are real words in Hakka and are not real words or morphemes in Mandarin. When recording these syllables, the native speakers were instructed to read a carrier sentence in their respective native languages with the target syllable. These Mandarin syllables were sourced from a speech dataset (Sinica COSPRO 08_M054) referenced in Tseng et al. (2005). The speaker was an adult male native speaker of Taiwanese Mandarin. The carrier sentence for the Mandarin syllables was “講話時動不動就會提到_” (whenever talking frequently mentions_). The speaker of Hakka syllables was a male native Hailu Hakka speaker who did not know Mandarin. These Hakka syllables were recorded in a phonological lab at Department of Hakka Language and Social Sciences and have been used in the study of Huang and Yu (2022). The carrier sentence for the Hakka syllables was “佢唸 _ 盡正” (He pronounces_very accurately). The syllables were then normalized to a duration of 350 ms and intensity of 70 dB using Praat. Speech waveforms and acoustic parameters of the stimuli are shown in Figure 2 and Table 1, respectively. Hakka syllable /so/ with 55-tone and Mandarin syllable /zo/ with 55-tone are very similar in F0 contour and direction. Likewise, Hakka syllable /so/ with 21-tone and Mandarin syllable /zo/ with 21-tone are very similar in F0 contour and direction.

**Table 1:** Voice onset time (VOT), the first three formant frequencies and F0 range for each stimuli.

	Hakka	Hakka	Mandarin	Mandarin
	/so 55/	/so 21/	/zu 55/	/zu 21/
VOT (ms)	122	122	94	99
F1 (Hz)	624	645	402	393
F2 (Hz)	1,030	1,059	851	764
F3 (Hz)	2,815	2,813	2,962	2,691
F0 range (Hz)	146–155	92–131	142–152	90–134

### 2.3 Procedure

Jacobsen and Schröger (2001) suggested that a control procedure would allow control of the state of refractoriness during the oddball paradigm. Accordingly, each participant was required to undergo four experimental blocks in the present study, consisting of a Mandarin-control block, Mandarin-MMN block, Hakka-control block, and Hakka-MMN block. During the experiment, participants were presented with spoken syllables of one language in each experimental block. In Mandarin blocks, participants heard the Mandarin Chinese syllable /zu/ with 55- and 21-tone. In Hakka blocks, the participants heard the syllable /so/ with 55- and 21-tone. Each experimental block comprised 500 trials. In the control blocks, syllables were randomly presented in 55-tone or 21-tone with equal probabilities (*p* = 0.5). In MMN blocks, the 55-tone and 21-tone syllables were randomly presented for 100 (*p* = 0.2) and 400 (*p* = 0.8) trials, respectively. The order of the experimental blocks was counterbalanced across participants. Each trial began with the presentation of a syllable lasting 350 ms (70 dB), followed by a 400-ms inter-trial interval. The syllables were presented using two loudspeakers. While participating in the experiment, participants watched a movie without sound or subtitles.

### 2.4 Data recording and preprocessing steps

EEG data were recorded using 32 Ag/AgCl electrodes (QuickCap, Neuromedical Supplies, Sterling, United States). The electrodes were online-referenced to the average of the left and right mastoids for offline analysis. The EEG was continuously recorded and digitized at a rate of 1,024 Hz, and the signal was amplified using a Grael 4 K EEG amplifier with a band-pass filter at DC–409 Hz. Electrode impedances were kept below 5 kΩ. Eye movements and blinks were monitored using supraorbital and infraorbital electrodes, and electrodes in the external canthi.

Instead of using conventional ERP analyses, we utilized the Hilbert-Huang transformation (Hsu et al., 2016; Huang et al., 1998) for the offline analysis, because HHT can provide better resolution. In brief, HHT is a two-step method for analysis of nonlinear and nonstationary signals. The first step is empirical mode decomposition (EMD). EMD is a data-driven, adaptive method that decomposes a signal into a finite number of intrinsic mode functions (IMFs) determined using an iterative sifting process that separates the signal into high and low frequency components. This decomposition method is adaptive and can automatically adjust to the signal’s frequency content. Once the signal is decomposed into IMFs, the instantaneous phase of each IMF can be calculated using the direct quadrature transform. The instantaneous frequency can then be obtained by calculating the time derivative of the instantaneous phase. This approach allows for better frequency resolution as it estimates the frequency content of the signal at each time point, rather than using methods such as the convolution integral method used in Fourier transform and wavelet transform. Therefore, HHT is useful in the analysis of non-linear and non-stationary signals.

In the present study, EEG data were analyzed in the following manner based on the procedure described by Hsu et al. (2016). Continuous EEG data were epoched with 100 ms pre-stimulus intervals and 600 ms post-stimulus intervals. The pre-stimulus interval (−100 to 0 ms) was used for baseline correction. Trials were rejected if they were contaminated by voltage variations larger than 100 μV in amplitude. We then decomposed each EEG segment into seven IMFs using the masked empirical mode decomposition (Quinn et al., 2021) and obtained event-related modes (ERMs) by averaging IMFs across trials (Al-Subari et al., 2015a,b). Similar to noise removal using filters, ERMs were obtained by summing the IMFs based on their instantaneous frequencies and then averaging across trials. The correlation between MMN components and frequency bands is well known, and Kalyakin et al. (2007) has suggested that EEG activity between 2 and 8 Hz reflects MMN. Previous studies of MMN responses using the HHT method confirmed that EMD can be used to analyze EEG signals and employ IMFs with frequencies ranging between 2 and 8 Hz to estimate MMN activity (Cong et al., 2009; Hsu et al., 2016). Therefore, this study focused on IMFs with frequencies between 2 and 8 Hz to extract MMN-related activities.

### 2.5 Statistical analysis

#### 2.5.1 Mass univariate cluster-based permutation tests

**Figure 3:**
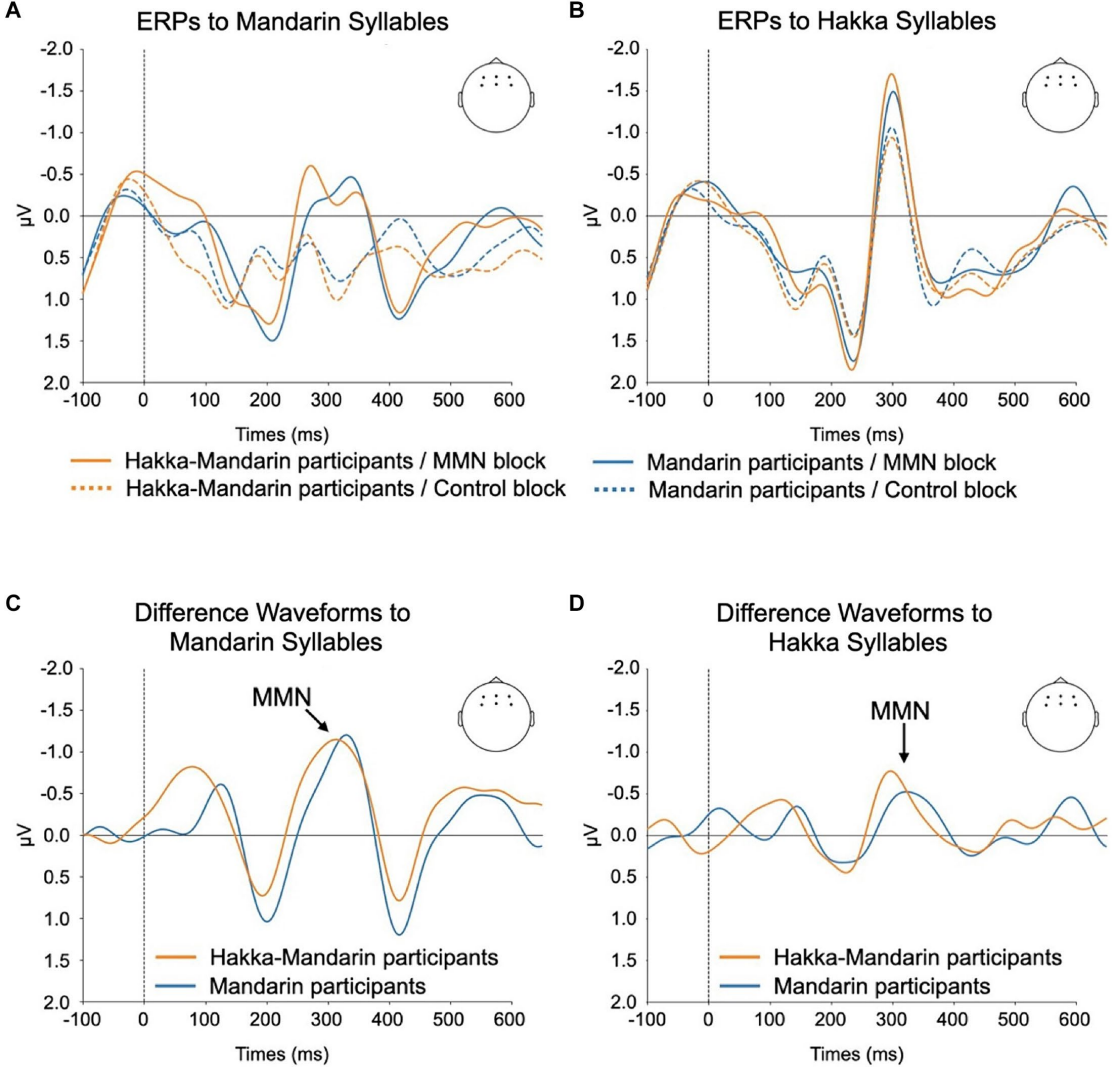
ERP waveforms elicited by speech stimuli averaged across six electrodes positioned on the anterior scalp. **(A)** displays the raw ERP waveforms in response to Mandarin syllables, while **(B)** illustrates the raw ERP waveforms elicited by Hakka syllables. **(C,D)** show difference waveforms for Mandarin and Hakka syllables, respectively. These difference waveforms are calculated by subtracting the ERP elicited by the 55-tone stimulus in the control block from the ERP of the identical stimulus in the MMN block.

In statistical analyses, the MMN effect was measured by comparing the ERPs to the deviant stimuli of the MMN block and ERPs to the same stimuli of the control (equal-probability) block. The grand-averaged waveforms for the 55-tone syllables in the MMN and control blocks are presented in Figures 3A,B. Through visual inspection of the data, a negative-going component peaking at around 300 ms was observed, and this component was more prominent in the waveforms of the MMN blocks than in those of the control blocks. We used mass univariate cluster-based permutation tests as recommended by Maris and Oostenveld (2007) to evaluate the significance of MMN activity (ERPs of the 55-tone stimuli in the MMN block versus ERPs of the same stimuli in the control block) of each participant group (Mandarin participants and Hakka-Mandarin participants) and each syllable (Mandarin syllable and Hakka syllable). This cluster-based nonparametric approach is recommended to control Type I error rates in electrophysiology experiments where precise latencies and scalp distribution are unknown *a priori*. The mass univariate cluster-based permutation tests were run using the Eelbrain package (version 0.39.8). The general procedure for the cluster-based test was as follows: a paired t-statistic (deviant minus control, one tail) was calculated at each time point and channel. Spatial–temporal clusters were then formed from test statistics that were contiguously significant (uncorrected *p* = 0.01) through time and channels. For each cluster, the cluster mass statistic was computed, which was the sum of all *t* values in the cluster. To determine the reliability of these clusters, the actually observed cluster-level test statistics were compared against the null distribution based on 10,000 random permutations of the condition labels.

In addition, we also analyzed the MMN activity obtained by calculating ERP difference waves through a subtraction method (Figures 3C,D). Specifically, the ERP elicited by the 55-tone stimulus in the control block was subtracted from the ERP of the identical stimulus in the MMN block. Then, the same mass univariate cluster-based permutation tests were applied to evaluate the effect of participant groups (MMN activity of Hakka-Mandarin participants minus MMN activity of Mandarin participants) and the effect of syllable types (MMN activity of Mandarin syllable minus MMN activity of Hakka syllable).

#### 2.5.2 Onset latency of MMN activity

Since cluster-based permutation tests on ERPs do not establish precise effect onsets or offsets (Sassenhagen and Draschkow, 2019), we tested the onset latency of MMN activity using the method described below. Difference waves in six electrodes (F3, FC3, Fz, FCz, F4 and FC4) distributed on the frontal scalp were used for the analysis of the onset latency. The latency of MMN activity was defined by finding the most negative peak from 200 ms to 400 ms in the difference wave (deviant condition minus control condition) and then working backward in the waveform until 50% of that peak voltage was reached. This approach has been highly recommended for quantifying the onset of an activity while applying to difference waves (Kiesel et al., 2008; Luck et al., 2009). For statistical analyses, the onset latency of MMN were analyzed with the linear mixed-effects model (Bates, 2005) with two random factors (random intercepts for participants and electrodes). The use of mixed-effects models with electrodes as a crossed random effect could omit strong effects mainly influenced by one electrode instead of all electrodes of interest. Language types (Hakka syllables versus Mandarin Syllable), participant groups (Hakka-Mandarin bilinguals versus Mandarin speakers), and their interaction were fixed factors. Latency was analyzed in R (Version 3.5.2) and RStudio (Version 1.1.463). The linear mixed-effects model was run using the “lmer” function as implemented in the lme4 package for R (Version 1.1–21). Reported *p*-values were calculated based on Satterthwaite’s method as implemented in the lmerTest package in R (Version 3.1–3). *Post hoc* comparisons were carried out using the “glht” function (the multcomp package, Version 1.4–15) with Bonferroni correction.

## 3 Results

### 3.1 MMN effect on ERPs in each language groups

**Figure 4:**
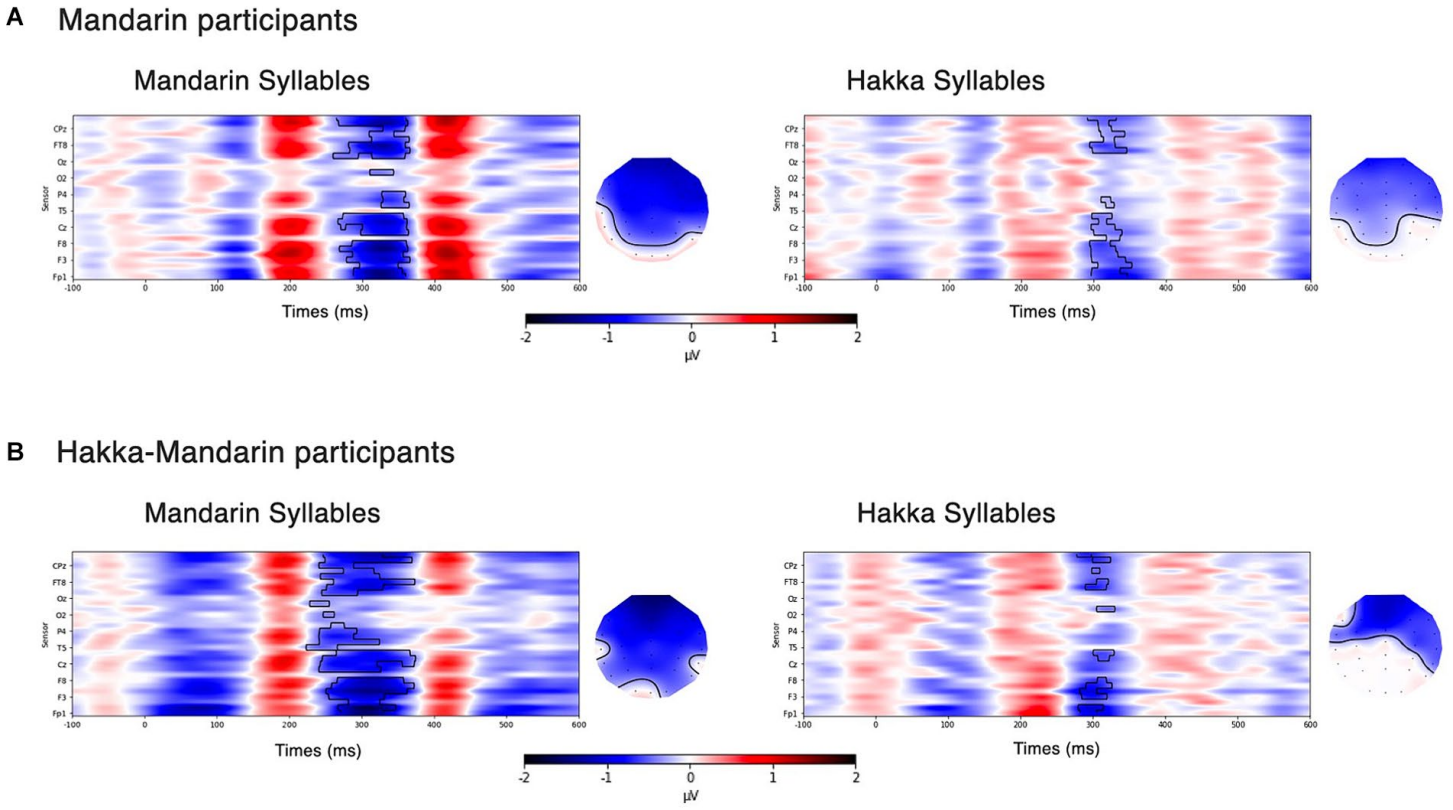
Visualization of difference waveforms across all channels. The black outline marks clusters in which ERPs in the MMN block and the control block differ significantly in time and across sensors, assessed by mass univariate cluster-based permutation tests. **(A)** Mandarin participants; **(B)** Hakka-Mandarin participants.

The results reported below were considered significant at a level of *p* < 0.05. Statistical results of the mass univariate analysis are shown in Figure 4. Overall, the results demonstrated that ERP amplitudes to 55-tones differed significantly between deviant and control conditions. Please note that these analyses do not establish precise effect onsets or offsets (Sassenhagen and Draschkow, 2019). Latencies were reported here as descriptive statistics. For Mandarin participants (Figure 4A), the MMN activity was observed between 260 and 366 ms (cluster-level *p* < 0.001) in the Mandarin syllable condition. On the other hand, their ERPs to Hakka syllables showed a significant spatial–temporal cluster between 292 to 353 ms (cluster-level *p* < 0.05). For Hakka-Mandarin participants (Figure 4B), the MMN activity was observed between 224 and 375 ms (cluster-level *p* < 0.001) in the Mandarin syllable condition, and their ERPs to Hakka syllables showed a significant spatial–temporal cluster between 280 to 331 ms (cluster-level *p* < 0.05).

### 3.2 Effects on MMN difference waveforms

While comparing the difference waves between Mandarin participants and Hakka-Mandarin participants, the mass univariate analysis did not reveal any significant spatial–temporal cluster. Finally, for the effect of syllable types, the mass univairate analysis also did not reveal any significant spatial–temporal cluster.

### 3.3 Effects on the latency of MMN activity

**Figure 5:**
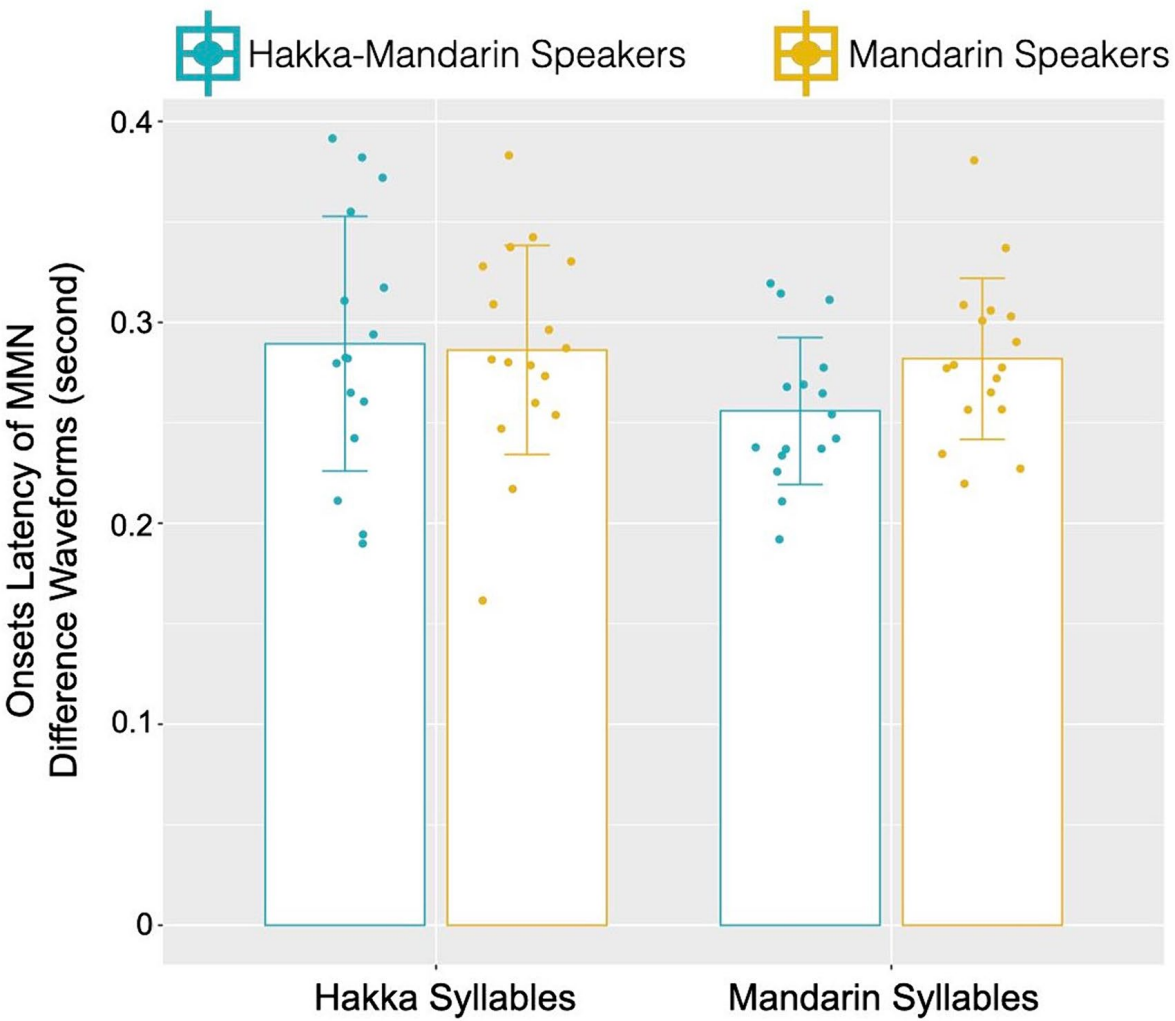
Means of onset latency of MMN waveforms averaged across participants and across electrodes of interests as a function of participant groups and syllable types. Error bars depict standard errors of means. Scatter dots illustrate individual datapoints.

The linear mixed-effects model analysis showed a significant main effect of syllable types (Hakka syllables versus Mandarin Syllable) on the latency of MMN (beta = −0.033, SE = 0.006, *p* < 0.0001), and the main effect of participant groups was not significant (beta = −0.003, SE = 0.012, *p* = 0.328). That is, Mandarin sounds elicited earlier latency of MMN (269 ms) than Hakka sounds (287 ms). In addition, there was a significant two-way interaction between syllable types and participant groups (beta = 0.029, SE = 0.009, *p* < 0.01), which showed that the simple main effect of participant groups was significant in Mandarin syllable condition (beta = −0.026, SE = 0.013, *p* < 0.05) but not in Hakka syllable condition (beta = 0.003, SE = 0.013, *p* > 0.05). Specifically (Figure 5), Hakka-Mandarin participants’ MMN latency (255 ms) was earlier than Mandarin participants’ MMN (281 ms) in the Mandarin syllable condition, and both participant groups showed similar MMN latency in the Hakka syllable condition (Hakka-Mandarin participants: 289 ms; Mandarin participants: 286 ms). Likewise, the simple main effect of syllable types was significant in Hakka-Mandarin participants (beta = 0.034, SE = 0.007, *p* < 0.01) but not in Mandarin participants (beta = 0.004, SE = 0.006, *p* > 0.05).

## 4 Conclusion

The present study found that ERPs to syllables in a deviant condition overall showed MMN activity. This finding was consistent with previous studies of MMN activity to tonal contrast which suggested that MMN activity is a robust index for the perception of pitch height in tonal language. As regarding the effect of tonal inventory, the working hypothesis for the present study was based on the assumption drawn in prior studies on vowel inventory. Specifically, Hacquard et al.’s (2007) study on MMN activity to vowel contrast demonstrated that the inventory organization of vowels would have an effect on perceived similarity which can be measured by the strength of MMN response. Building on the findings of the impact of phoneme inventory size on perceptual ability, one could posit that a similar correlation would exist between the complexity of a tone system in tonal languages and the perceptual abilities of its speakers. Note that the tonal inventories of Hailu Hakka are greater than those of Mandarin Chinese. Therefore, it was hypothesized that the MMN of Hakka-Mandarin speakers would be larger or earlier than that of Mandarin participants.

Although the present results for amplitude data did not show a significant difference between Hakka-Mandarin participants and Mandarin participants, the analysis of onset latency did show a significant difference between participant groups in the Mandarin syllable condition, and the pattern of MMN latencies aligned with the hypothesis. Our results showed that Hakka-Mandarin speakers demonstrated earlier MMN latency than Mandarin speakers in processing Mandarin syllables. In the literature, MMN is viewed as a reflection of an automatic, pre-attentive auditory processing mechanism, so the present observation of delayed MMN in Mandarin participants provides insights into the speed and efficiency of their auditory change detection. On the other hand, both participant groups showed similar MMN latency in the Hakka syllable condition. It may appear somewhat unexpected that the effect of tonal inventory was found only for the Mandarin syllable condition. The interaction between participants’ language background (Hakka-Mandarin speakers versus Mandarin speakers) and types of syllables (Mandarin syllable /zu/ and Hakka syllable /so/) on the latency of MMN might indicate that other factors also play a crucial role on MMN responses to tonal contrast. One possibility is that the sonority of syllable also had an effect here. Since the stimuli used in the presented experiment were CV syllables without consonant clusters, it is assumed that the quality of the vowel would be a factor that influence the perceived sonority of the syllable. In the sonority hierarchy, mid vowels like /o/ are often considered to be more sonorous than high vowels like /u/. There is evidence suggesting that the perception of suprasegmental features, such as tonal contrasts, is more accurate in regions of higher sonority (Niebuhr, 2007; Zhang, 2002). Given this, the Hakka syllable /so/ could result in similar MMN latencies for both Hakka-Mandarin speakers and Mandarin speakers because the high sonority enhances the robustness of its auditory processing. On the other hand, individuals with multiple phonemic inventories may have more refined auditory processing capabilities, which could affect their MMN responses. Therefore, the experiences with multiple phonemic inventories could enhance Hakka-Mandarin speakers’ perceptual ability for the less sonorous syllable /zu/, leading to earlier MMN latencies compared to Mandarin speakers.

One might suggest that the observed tonal inventory effect could be attributed to a training effect, given that Hakka-Mandarin speakers are enrolled in undergraduate or graduate programs where Hailu Hakka is the primary language of instruction. However, if training were a concern, it is important to consider the relationship between lexical familiarity and MMN activity to words. Since bilingual individuals divide their language use between two languages, their frequency of use in each language is typically lower than that of monolinguals who use a single language. This reduced frequency of use would result in lower familiarity with vocabulary in each language. Studies using vocabulary fluency tasks have indicated that bilingual adults and children often experience difficulty in word production across languages (for a review, see Bialystok et al., 2009). Given that lexical familiarity also influences MMN to spoken words (Pulvermüller and Shtyrov, 2006), it could be expected that Hakka-Mandarin speakers might exhibit reduced or delayed MMN activation compared to Mandarin speakers. That is, if the training effect were significant, Hakka-Mandarin speakers would likely show reduced or delayed MMN responses due to lower familiarity. The fact that they do not exhibit such patterns suggests that the observed effects may be due to tonal inventory and language background.

It is worth noting that the Hakka syllables /so/ used in the present experiment are not real syllables in Mandarin Chinese, and the Mandarin syllables /zu/ are not real syllables in Hakka. By using syllables that are unique to each language, it becomes easier to attribute differences in perceptual processing to the participants’ language background. For example, Kuo et al. (2016) investigated the effect of phoneme inventory on speech perception, and their results showed that bilingual children have a heightened phonological awareness while processing consonants that were shared between languages. Their results suggested that bilingual children would benefit from experiencing phonological segments in more variable contexts. This enhanced experience allows bilingual children to better dissociate phonological segments from their contexts, giving them an advantage in processing shared onsets. In the present study, the results demonstrated the effect of tonal inventory in processing unique syllables. Our approach avoids explanations based on shared phonological representations in richer and more variable contexts, which could also enhance speech perception abilities. Nevertheless, the present results were also in line with Kuo et al. (2016)’s finding that bilingual individuals develop a heightened awareness of language structure due to their exposure to a wider variety of phonemic contrasts.

Another observation to mention is that Mandarin speakers exhibited MMN activity to Hakka syllable /so/ which is not a real word or real morpheme in Mandarin Chinese. We speculated that this finding could reflect the organization of the phonological representation in tonal language speakers, particularly demonstrating that tonal representations are stored and processed parallel to segmental features (Malins and Joanisse, 2010). That is, MMN is a brain response to violations of a rule established by a sequence of auditory stimuli. Therefore, in the context of language, MMN to tonal changes is associated with how elements of syllables are processed and stored. The present study’s finding that Mandarin speakers exhibit MMN to tonal changes in a non-native syllable suggests that their phonological system is attuned not only to the segmental features of their native language but also to its tonal aspects. The tonal patterns used in the present experiment are native tones, which Mandarin speakers are familiar with. This familiarity might allow the mapping of these tonal patterns onto existing tonal exemplars in their lexicon, even though the segmental part of the stimuli is non-native. This finding is consistent with other studies on perceptual integration of tones and syllables in non-native speech perception (Choi and Tsui, 2023; Lin and Francis, 2014).

Although the present study provides implications of the tonal inventory effect on speech perception, some issues still need to be investigated in the future. The present study did not utilize tools such as the Language History Questionnaire (LHQ) to gather detailed information about the participants’ linguistic backgrounds (Li et al., 2020). Using an LHQ in future studies would allow for a more comprehensive profile of each participant’s language experience, which can help to provide a richer context for interpreting the results. Secondly, this study did not account for the participants’ musical backgrounds, which could have an influence on auditory processing and speech perception. Musical training is known to enhance auditory discrimination skills. Future research should include an assessment of the participants’ musical backgrounds to determine whether musical experience contributes to differences in MMN responses and to isolate the effects of linguistic experience from those of musical training. Finally, while MMN activity is associated with the diversity of tonal representations, a caveat emerges when considering the influence of the size of the tonal space. This aspect remains ambiguous, particularly in light of speech production studies that have not found notable differences in the fundamental frequency (F0) space across tonal languages (Kuang, 2013). Therefore, while it is clear that MMN activity is responsive to variations in tones, the extent to which the size of the tonal space itself affects this neural response has not been well-established.

In conclusion, this study provides valuable insights into how speakers of languages with more intricate tonal systems, such as Hailu Hakka with its seven distinct tones and variations in pitch height, contour, and duration, exhibit enhanced perceptual sensitivity to tonal contrasts compared to speakers of languages with simpler tonal systems. These findings should be interpreted with caution as the language history data might be insufficient. Despite of this limitation, the results support the idea that a linguistically rich environment shapes perceptual processes and fosters greater perceptual acuity.

## Data availability statement

The raw data supporting the conclusions of this article will be made available by the authors, without undue reservation.

## Ethics statement

The studies involving humans were approved by Human Subject Research Ethics Committee of National Taiwan University. The studies were conducted in accordance with the local legislation and institutional requirements. The participants provided their written informed consent to participate in this study.

## Author contributions

C-HH: Writing – review & editing, Writing – original draft, Visualization, Supervision, Resources, Project administration, Methodology, Funding acquisition, Conceptualization. T-HC: Writing – review & editing, Software, Methodology, Formal analysis, Data curation. W-JH: Writing – review & editing, Writing – original draft, Visualization, Validation, Resources, Methodology, Funding acquisition, Conceptualization.
